# Antidepressant Use and Lung Cancer Risk and Survival: A Meta-analysis of Observational Studies

**DOI:** 10.1158/2767-9764.CRC-23-0003

**Published:** 2023-06-12

**Authors:** Eunkyung Lee, Yongho Park, David Li, Alice Rodriguez-Fuguet, Xiaochuan Wang, Wen Cai Zhang

**Affiliations:** 1 Department of Health Sciences, College of Health Professions and Sciences, University of Central Florida, Orlando, Florida.; 2 College of Medicine, University of Central Florida, Orlando, Florida.; 3 Department of Cancer Division, Burnett School of Biomedical Sciences, College of Medicine, University of Central Florida, Orlando, Florida.; 4 School of Social Work, College of Health Professions and Sciences, University of Central Florida, Orlando, Florida.

## Abstract

Recent preclinical studies have linked antidepressants (AD) to their potential anticancer effects in multiple cancers, but the impact on lung cancer remains unclear. This meta-analysis examined the associations between ADs and lung cancer incidence and survival. The Web of Science, Medline, CINAHL, and PsycINFO databases were searched to identify eligible studies published by June 2022. We conducted a meta-analysis using a random-effects model to compare the pooled risk ratio (RR) and 95% confidence interval (CI) in those treated with or without ADs. Heterogeneity was examined using Cochran *Q* test and inconsistency *I*^2^ statistics. The methodologic quality of the selected studies was assessed using the Newcastle-Ottawa Scale for observational studies. Our analysis, including 11 publications involving 1,200,885 participants, showed that AD use increased lung cancer risk by 11% (RR = 1.11; 95% CI = 1.02–1.20; *I*^2^ = 65.03%; *n* = 6) but was not associated with overall survival (RR = 1.04; 95% CI = 0.75–1.45; *I*^2^ = 83.40%; *n* = 4). One study examined cancer-specific survival. Subgroup analysis showed that serotonin and norepinephrine reuptake inhibitors (SNRIs) were associated with an increased lung cancer risk by 38% (RR = 1.38; 95% CI = 1.07–1.78; *n* = 2). The quality of selected studies was good (*n* = 5) to fair (*n* = 6). Our data analysis suggests that SNRIs were associated with an elevated risk of lung cancer, raising concerns regarding the use of AD treatment in patients vulnerable to lung cancer. The effects of ADs—particularly SNRIs—and their interplay with cigarette use and lung cancer risk in vulnerable patients merits further study.

**Significance:**

In this meta-analysis of 11 observational studies, we found evidence of a statistically significant association between the use of certain ADs and lung cancer risk. This effect merits further study, particularly as it relates to known environmental and behavioral drivers of lung cancer risk, such as air pollution and cigarette smoke.

## Introduction

Lung cancer is the second most common cancer and the leading cause of cancer-related deaths worldwide, accounting for approximately 11% of newly diagnosed cancers and 18% of cancer deaths in 2020 (1). Current lung cancer treatments include surgery, chemotherapy, radiotherapy, and targeted therapy depending on several factors including the stage and the type of lung cancer (2). Despite these many treatments available, the average 5-year relative survival rate for lung cancer is 22%, lower than many other cancers, and ranges from 7% for those with small cell lung cancer (SCLC) to 26% for non–small cell lung cancer (NSCLC; ref. 3). As the low survival rate arises partly from difficulties in the early detection of lung cancer, a better understanding of patients’ clinical features and risk factors could facilitate more timely intervention leading to improved survival (4).

Antidepressants (AD), which were used by 13.2% of U.S. adults in the past 30 days during 2015–2018 (5), have been reported to have inconsistent associations with lung cancer risk. Previous *in vitro* and preclinical studies have suggested that ADs may have anticarcinogenic or tumor-suppressive effects. Cordero and colleagues showed that amitriptyline, a tricyclic antidepressant (TCA), had antitumor effects on human tumor cell lines, including lung cancer, cervical cancer, and hepatoma (6). When compared with three commonly used ADs, amitriptyline produced elevated levels of tumor cell damage by increasing the levels of intracellular reactive oxygen species while inhibiting antioxidant formation in the tumor cells (6). Another FDA-approved TCA, imipramine, suppressed tumor progression in glioma cell lines and mouse models by inhibiting the activity of the oncogenic yes-associated protein 1 (YAP1) (7). Selective serotonin reuptake inhibitors (SSRIs), sertraline and fluoxetine, suppressed the growth of NSCLC, gastric cancer, melanoma, and hepatocellular carcinoma by inhibiting the mTOR activity (8–12).

While these tumor-suppressing effects of ADs are encouraging, tumor-promoting results have also been reported in the literature. For example, amitriptyline and fluoxetine promoted the development of fibrosarcoma and melanoma in rodents at clinically relevant doses (13). However, conflicting results have been obtained from epidemiologic studies. A meta-analysis for colorectal cancer reported a reduced risk with TCA use (14), while another meta-analysis for ovarian cancer showed no associations with either SSRI or TCA use (15). In addition, the effect of these ADs on lung cancer risk in humans is still unclear, and none of the studies evaluated the association between serotonin and norepinephrine reuptake inhibitors (SNRIs) and lung cancer outcomes.

Considering the growing use of ADs for the treatment of other conditions such as chronic pain (16) and smoking cessation (17) as well as depression and the high prevalence of depression among patients with lung cancer (18, 19), we performed a meta-analysis to summarize the currently available evidence on the relationship between AD use and lung cancer risk in the general population, and survival in patients with lung cancer.

## Materials and Methods

This meta-analysis was performed in accordance with the Preferred Reporting Items for Systematic Reviews and Meta-Analyses (PRISMA) and was registered in the International Prospective Register of Systematic Reviews (PROSPERO; University of York; York, United Kingdom) platform with the registration number CRD42022350719. As this study is a summary of previously published studies, no ethical review is required.

### Search Strategy

A comprehensive search was conducted using key terms to maximize the identification of the studies examining the association between AD use and lung cancer incidence and survival. Online search platforms, EBSCOhost (Medline, CINAHL, and PsycINFO) and Web of Science, were searched from inception to June 2022. The search terms included: lung cancer or lung neoplasms or lung tumor or lung adenocarcinoma or NSCLC or non–small cell lung cancer or SCLC or small cell lung cancer AND antidepressant(s) or anti-depressant(s) or AD medication or SSRI or selective serotonin reuptake inhibitor(s) or SNRI or serotonin-norepinephrine reuptake inhibitor(s) or TCA or tricyclic AND mortality or mortality rate or death or death rate or survival rate or survival outcomes or clinical outcomes or incidence or risk. Filters included human studies and articles published in peer-reviewed journals. We also reviewed the reference lists of the included studies and prior meta-analyses and reviews to identify additional publications that could be eligible. The full search strategies are shown in Supplementary Table S1.

### Inclusion and Exclusion Criteria

The search results from four databases were first uploaded to Covidence software (Veritas Health Innovation; ref. 20), and two reviewers independently screened articles according to the Participants, Intervention/Exposure, Comparison, Outcome, and Study design guidelines outlined in Supplementary Table S2. The population included adults (adults without cancer for lung cancer risk studies or adult lung cancer survivors for survival outcomes) with exposure to AD use. The outcomes included lung cancer incidence or survival from observational studies, including case–control or cohort studies. We excluded review literature, meta-analysis, newsletters, conference abstracts, and animal studies. We also excluded literature evaluating survival relating to AD use in the general population, not from lung cancer survivors.

### Data Extraction and Methodologic Quality Evaluation

The quality of the studies was assessed using the Newcastle-Ottawa Scale for observational studies (21). This tool evaluates the quality of case–control and cohort studies on the following three domains regarding (i) the adequacy of the recruitment and selection of study participants, (ii) the comparability of comparison groups, and (iii) the ascertainment of exposure and outcomes. Studies that received a score of 7–9 were considered good, 4–6 as fair, and 0–3 as poor.

Two reviewers extracted the data using a predesigned data extraction form created by authors on Covidence. Disagreements were resolved through discussion by consensus with all authors. The following data were extracted from each study. Only information relevant to lung cancer was extracted when the analysis included multiple cancers.

- Study characteristics: title, first author, year of publication, country of study, study design, study name or source population, number of participants, follow-up years or study period, and study objectives (purposes).
- Population characteristics: eligibility criteria, age (mean or distribution), gender, smoking, depression, lung cancer histology, and treatments received.
- Exposure: types (SSRI, SNRI, TCA, Other), timing (prediagnosis or postdiagnosis), ascertainment methods (medical records, prescription database, or questionnaire), and comparison (current use, past use, nonuse, or by-dose increment) of AD use.
- Outcome:
  - Endpoints included incidence or survival, the total number of outcomes (overall or per group, if available), and ascertainment methods (medical records, pathologic exams, cancer registry, National Death Index, or survey).
  - Covariates included in the multivariable model.
  - Overall findings from the study, including odds ratio (OR), risk ratio (RR), or hazard ratio (HR) and a 95% confidence interval (CI) comparing two treatment groups or by dose increment (if available).

### Statistical Analysis

The meta-analysis was performed by combining the multivariable-adjusted OR/RR/HR of the association between AD use and lung cancer from each study using the Bayesian random-effects pooling model. A Bayesian approach reflects uncertainty in the estimation of between-study heterogeneity and is a better approach when the number of studies in the meta-analysis is small compared with a classic approach using the DerSimonian–Laird method (22). When the study reported the OR/RR/HR for each AD, a combined effect and 95% CI for that study were first obtained using the fixed-effects pooling model. The analysis was conducted separately for lung cancer risk and survival to evaluate whether the associations with AD use were the same for two different outcomes.

Subgroup analysis was conducted to investigate potential sources of heterogeneity. First, the extent to which AD type was associated with lung cancer risk or survival was assessed. Second, study characteristics (study location, design, quality, and comparison groups) were evaluated to examine whether the associations were consistent for different study conditions. Third, AD use with respect to lung cancer diagnosis was evaluated to assess whether prediagnosis and postdiagnosis AD use had a differential association with survival. Heterogeneity between studies was quantitatively assessed using the Cochran *Q* test and inconsistency *I^2^* test. Egger test and the funnel plot were used to determine the potential existence of publication bias in meta-analysis. Statistical analyses were performed using the Comprehensive Meta-Analysis software (version 3, Biostat Inc.) and Stata – Release 17 (StataCorp). A two-tailed *P* < 0.05 was considered statistically significant.

### Data Availability

The data generated in this study for the meta-analyses are available upon request from the corresponding author. The data generated in this study, apart from the data used for the meta-analyses, are available within the article.

## Results

### Literature Search Results

**FIGURE 1:**
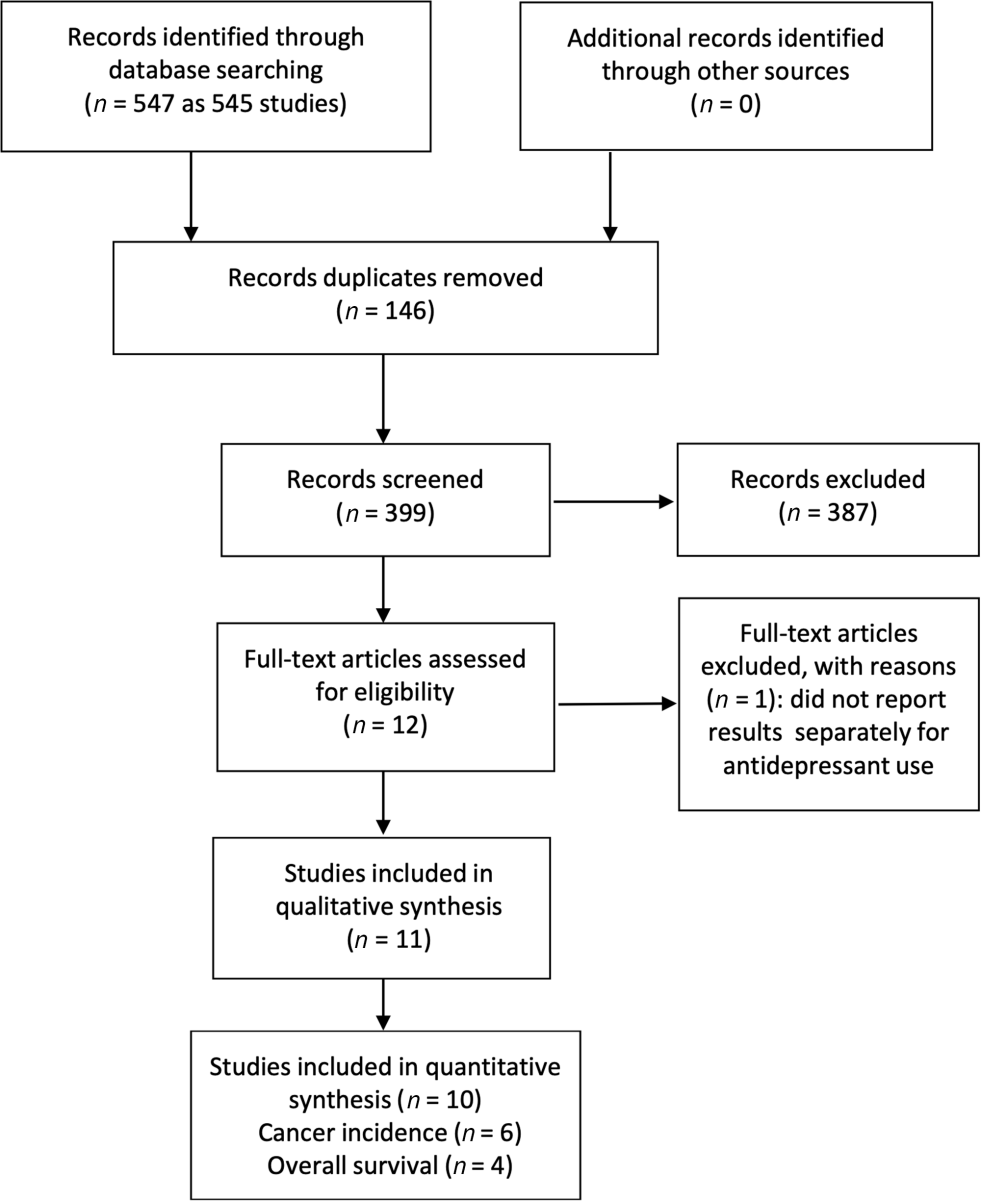
Flow diagram of the study selection process.

We screened 545 abstracts, 12 of which were reviewed in full for eligibility. We excluded one full-text publication due to no results being reported separately for AD use (23), leaving 11 for data extraction and quality assessment. The detailed steps of the systematic search and selection processes are shown in Fig. 1 of a PRISMA flow chart.

### Characteristics of Included Studies

The characteristics of included studies are summarized in Table 1. Four studies (24–27) were conducted in the United Kingdom, two studies (28, 29) in the United States, two studies (30, 31) in Finland, and one in Hungary (32), Israel (33), and Taiwan (34), respectively. Seven studies (27–33) used a retrospective cohort study design, while four studies (24–26, 34) used a case–control study design. The sample size varied from 174 patients with lung cancer in a single medical center study (28) to 418,588 pairs in a population-based retrospective cohort study (31). The proportion of male participants also varied from 35.3% (33) to 100% (30), and smoking prevalence ranged from 0.15% in a population-based lung cancer incidence study (34) to 92% in a clinic-based lung cancer survival study (29). Only five studies (24, 25, 30, 33, 34) reported the prevalence of depression in their study sample, which ranged from 3.5% in a population-based case–control study (34) to 31% in a population-based study but only including those with at least one AD purchase (33). Only three studies specified the histology of the lung cancer cases they included: one study (28) examined the association between ADs and overall survival among patients with NSCLC, another study (32) assessed the association with overall survival among those with SCLC, and the last one (29) measured cancer-specific survival among those with adenocarcinoma, squamous cell carcinoma, large cell carcinoma, SCLC, or NSCLC.

**TABLE 1:** Characteristics of studies of lung cancer risk and survival associated with AD use included in meta-analysis

Author Year Country	Study design No. of participants Source population Study period or follow-up	Eligibility Mean age (SD) or range	Men (%) Ever smokers (%) Depression (%) Histology	Types of drugs Timing and ascertainment of AD use Comparisons	Outcome (cases) Ascertainment of outcome	Study purpose	Main findings OR/RR (95% CI)	Covariates	Notes
Knekt 1996 (30) Finland	Retrospective cohort study 3,245 (35, 3,210) The Mini-Finland Health Survey 1978–1991 (max 14 years)	Finnish adults ages 30 years and over were randomly selected for the survey and interview 30–95	100% 70.5% 9% Unknown	Unknown Before diagnosis. Interview Current use vs. no use	Incidence (70; 2, 68) Finnish Cancer Registry	To examine the association between depression and subsequent lung cancer occurrence	Depressiveness was associated with the incidence of lung cancer. AD use was not associated with male lung cancer incidence. AD: 2.00 (0.49–8.15)	Age	
Walker 2011 (24) United Kingdom	Matched case–control study 19,051 (6,537, 12,514) GPRD ≥5 years	Cases were anyone ages 18 or above with a recorded diagnosis of lung cancer and >5 years of follow-up. Controls without cancer were matched on age, sex, practice site with a 1:2 ratio 71.0	61.7% vs. 61.2% 38.2%^a^ 23.7%^a^ Unknown	TCA 1 year before diagnosis Medical records Use vs. no use	Incidence (6,537) Medical records	To investigate relations between cancer and TCA	15.0% of cases and 12.2% controls were TCA users. A slight increase in risk of lung cancer from TCA use was seen. TCA: 1.14 (1.02–1.28)	Smoking, diagnosis of depression, alcohol use, and BMI	
Tsai 2019 (34) Taiwan	Matched case–control study 228,907 (39,001, 189,906) NHIRD 1999–2008	Cases were new onset lung cancer in 1999–2008 and age- and gender-matched controls were those without cancer with a 1:5 ratio ≤40: 10.7% vs. 10.8% 41–50: 22.6% vs. 22.8% 51–60: 27.6% vs. 27.8% 61–70: 24.6% vs. 24.6% 71–80: 14.5% vs. 14.1%	53.6% vs. 53.4% 0.3% vs. 0.15% 4.0% vs. 3.52% Unknown	SSRI; SNRI; TCA; MAOI, mirtazapine, trazodone and bupropion Before diagnosis Medical records; Prescription records Cumulative dose increment	Incidence (39,001) Medical records + Catastrophic Illness Certificate	To evaluate association between AD and lung cancer risk	26.8 (22.5%) of cases (controls) were exposed to any types of antidepressants. AD prescription did not elevate the risk for lung cancer with exception of bupropion at high exposure levels. SSRI: 0.99 (0.87–1.13) SNRI: 1.42 (0.99–2.04) TCA: 0.86 (0.71–1.03) MAOI: 0.95 (0.82–1.11) Mirtazapine: 0.83 (0.29–2.43) Trazodone: 1.31 (0.95–1.81) Bupropion: 4.81 (1.39–16.71)	Urbanization, income, hypertension, T2DM, hypercholesterolemia, COPD, pneumonia, pulmonary TB, asthma, coal worker pneumoconiosis, asbestosis, toxic effect of arsenic, HIV infection, smoking, and medication	Population based, matched study. NHIRD covers >99% of population.
Toh 2007 (25) United Kingdom	Nested case–control study 14,336 (4,336, 100,00) THIN database 1995–2005	Cases were new onset of primary lung case in 1995–2005. Controls were randomly sampled from the same study cohort and matched on age, sex, and diagnosis year 40–85	61.3% vs. 60.5% 70.3 vs. 32.4% 17.5% vs. 12.7% Unknown	SSRI; TCA 1 year before diagnosis Prescriptions Recent use, past use, and no use; high dose, low dose	Incidence (4,336) Medical records	To evaluate the effect of AD use on lung cancer risk	18.7 (14.2%) and 10.1 (7.5)% of cases (controls) were exposed to TCAs and SSRIs, respectively. Long-term TCA use was not associated with a reduced risk of lung cancer while long-term SSRI use might be associated with a lower risk. *Recent use vs. no use* TCA: 1.23 (0.96–1.58) SSRI: 0.59 (0.41–0.86) *Past use vs. no use* TCA: 0.98 (0.83–1.14) SSRI: 1.07 (0.86–1.33) High dose vs. no use among recent users TCA: 1.52 (1.07–2.16) SSRI: 1.25 (0.45–3.50)	Age, sex, year of diagnosis, use of other types of ADs, smoking, smoking cessation intervention, history of depression and anxiety, health care utilization indicators, COPD, and use of antibiotics	
Haukka 2010 (31) Finland	Retrospective cohort study 837,176 (418,588 pairs) SII- FCR linked data 4.01 years	AD users were individuals who (i) have purchased at least one prescription of AD between 1998 and 2005, (ii) had not purchased any AD between 1995 and 1997 and (iii) had no cancer diagnosis at the date of the first purchase. AD nonusers were randomly selected from the general population and were without cancer. Females: 48.0 (IQR: 35.0–58.0)^a^ Males: 45.8 (IQR: 34.0–56.0)^a^	39.8% NR NR Unknown	SSRI; non-SSRIs Before diagnosis Prescription database Cumulative dose increment	Incidence (1,731) Finnish Cancer Registry	To study the association between exposure to AD medication and the subsequent risk of 20 cancers	The authors did not find clear evidence of beneficial nor harmful association between AD use and cancer. *High dose vs. no use* SSRI: 1.08 (0.93–1.26) Non-SSRI: 1.36 (1.09–1.69)	Age, sex, length of follow up, and cumulative use of SSRI and non-SSRI	Data were extracted for lung cancer cases only.
Boursi 2015 (26) United Kingdom	Nested case–control study 93,616 (19,143, 74,473) THIN database 6.2–6.3 years	All people receiving medical care from 1995 to 2013 from a THIN practitioner were eligible. Cases were new onset of primary cancer and controls were selected based on incidence-density sampling with a 1:4 ratio. 71.2 (10.5) vs. 71.0 (10.6)	57.2% vs. 57.1% 77.1% vs. 44.3% NR Unknown	SSRI; SNRI; TCA More than 1 year before diagnosis Medical records Current use, past use, and no-use; cumulative duration of therapy	Incidence (19,143) Medical records	To evaluate the association between exposure to SSRIs, TCAs and SNRIs and the five most common solid tumor (lung, colorectal, breast, prostate, and melanoma)	There was a higher risk for lung cancers among individuals currently treated with SSRI or TCA with initiation of therapy more than one year before index date. However, there was no association between cumulative duration of AD therapy and cancer risk. *Recent use vs. no use* SSRI: 1.27 (1.16–1.38) SNRI: 1.01 (0.67–1.52) TCA: 1.45 (1.31–1.6) *Past use vs. no use* SSRI: 1.14 (1.06–1.22) SNRI: 1.77 (1.31–2.38) TCA: 1.06 (0.98–1.15) Treatment >3 years vs. no use SSRI: 1.24 (1.09–1.42) SNRI: 1.07 (0.58–1.97) TCA: 1.54 (1.33–1.78)	Age at index, sex, practice site, duration of follow-up, smoking history (ever or never), diabetes mellitus, and COPD	Controls were matched on age, sex, practice site, and duration of follow-up.
Abdel Karim 2019 (28) United States	Retrospective cohort study 174 (34, 140) University of Cincinnati Medical Center database 40 months	Patients with NSCLC between Jan 2004 and Dec 2014. Patients who received antidepressants in the form of TCAs, SSRIs, and other drugs were compared with those who received no antidepressants. 61 (IQR: 55–69)	55.7% (35% vs. 61%) NR NR NSCLC, stage 1–4	SSRI; TCA; Other After diagnosis Medical records Use, no use	All-cause mortality (84; 16, 68) Medical records	To evaluate the survival benefit of TCAs in lung cancer patients based on systematic computational drug repositioning data	No benefits of using anti-depressants in NSCLC patients AD: 1.21 (0.67, 2.20) TCA: 6.72 (1.59, 28.48) SSRI: 0.98 (0.47, 2.05) Other: 1.31 (0.57, 3.02)	Age, sex, and stage	All patients received platinum-based chemotherapy.
Shoval 2019 (33) Israel	Retrospective cohort study 1,183 National CHS database Median 25 months	Those who purchased AD at least one time during study period. 90% were > 40^a^	35.3%^a^ 29.0%^a^ 31% Unknown	Unknown After diagnosis Medical records + pharmacy data Good (>80%), moderate (50%–80%), poor (20%–50%) and nonadherence (<20%)	All-cause mortality (561) Ministry for Interior Affairs (governmental vital statistics)	To investigate the association between AD adherence and mortality in people with cancer	Greater adherence to AD was associated with decreased risk of all-cause mortality during the 4-year follow-up period after adjusting for multiple risk factors. AD: 0.79 (0.64, 0.98)	Age, sex, smoking status, socioeconomic status, and Charlson comorbidity index	Adherence was defined as the period of AD purchased divided by study participation duration (months). CHS covers ∼53% of population.
Lohinai 2016 (32) Hungary	Retrospective cohort study 876 National Koranyi Institute of Pulmonology Median 6.5 months (range: 0–150)	Consecutive patients with stage 4 SCLC in the clinic between 2000 and 2013 Median 61 (range: 33–86)	58% NR NR SCLC, stage 4	SSRI; TCA NR Medical records After diagnosis Use, no use	All-cause mortality (868) Medical records	To evaluate whether FDA-approved anti-SCLC activity in preclinical models is associated with survival benefit in a large, well-defined cohort of metastatic SCLC patients from a single institution	In the multivariate setting among therapies only RT is an independent prognosticator for increased overall survival. SSRI: 0.78 (0.52, 1.16) TCA: 0.96 (0.40, 2.27)	None	Only 5 used TCA and 20 used SSRIs. Patients were treated with chemotherapy and RT.
Boursi 2018 United Kingdom	Retrospective cohort study 1,224 (713, 511) THIN 7.1–7.4 years	All patients receiving medical care from 1995 to 2013 from a THIN practitioner. Each cohort study included only individuals with a specific cancer during the follow-up period and at least one prescription for SSRIs more than 1 year prior to cancer diagnosis 69.9 (10.6) vs. 71.2 (10.1)	42% 84.2% NR Unknown	SSRI More than 1 year before and after diagnosis Medical records Continuous use, past use	All-cause mortality (930; 578, 352) Medical records	To evaluate the impact of chronic use of SSRIs on survival of cancer patients with the five most common solid tumors (lung, colorectal, breast, prostate, and melanoma)	Decreased survival among cancer patients with continuous SSRI therapy was observed. The risk did not increase with treatment duration. SSRI: 1.51 (1.21, 1.72)	Age, sex, duration of follow-up from first SSRI prescription to cancer diagnosis date, smoking, COPD, previous respiratory infections, and diabetes	
Zingone 2017 (29) United States	Retrospective cohort study 1,097 (207, 890) NCI-MD lung cancer case–control study 5 years	Patients with pathologically confirmed lung cancer from the greater metropolitan area of Baltimore between 1998 and 2010 65.8 (10.5)	51% 92% NR 46% Adenocarcinoma, 26% Squamous cell carcinoma, 2% large cell carcinoma, and 26% other including NSCLC and SCLC	SSRI, SNRI, TCA, NDRIs, SRIs, NSSRIs, MAOIs 3 months before diagnosis Questionnaire/ survey Use, no use	All-cause death (698), Cancer-mortality (647; 110, 537) National Death Index	To examine the association between AD use and survival in lung cancer	TCA is associated with prolonged lung cancer survival. *Cancer-specific mortality* AD: 0.68 (0.49, 0.95) TCA: 0.40 (0.17, 0.92) SNRI: 1.63 (0.56, 4.78) SSRI: 0.72 (0.45, 1.17)	Age, race, gender, smoking status, pack-years of smoking, stage, histology, income, education level, and drug indication	

Abbreviations: AD, antidepressant; CHS, Clalit Health Services; COPD, chronic obstructive pulmonary disease; FCR, The Finnish Cancer Registry; FDA, Food and Drug Administration; GPRD, General Practice Research Database; HIV, human immunodeficiency virus; IQR, interquartile range; MAOIs, monoamine oxidase inhibitors; NCI-MD, National Cancer Institute – Maryland; NHIRD, National Health Insurance Research Database; NR, not reported; NSCLC, non–small cell lung cancer; RT, radiotherapy; SCLC, small cell lung cancer; SII, Social Insurance Institution; SNRI, serotonin-norepinephrine reuptake inhibitor; SSRI, selective serotonin reuptake inhibitor; T2DM, type 2 diabetes mellitus; TB, tuberculosis; TCA, tricyclic antidepressants; THIN, The Health Improvement Network.

^a^Presented values are for all study samples, including lung cancer.

#### AD Use

Regarding the types of ADs used, two studies (30, 33) did not specify them. One study (24) focused on TCAs, another study (27) focused on SSRIs, and all other studies (25, 26, 28, 29, 31, 32, 34) included multiple types and evaluated the association of ADs separately; seven studies evaluated SSRIs (25, 26, 28, 29, 31, 32, 34), three studies examined SNRIs (26, 29, 34), and six studies assessed TCAs (25, 26, 28, 29, 32, 34). Information on AD use was obtained from interviews/questionnaires (*n* = 2; refs. 29, 30) or medical/prescription records (*n* = 9; refs. 24–28, 31–34). Seven studies (24–26, 29–31, 34) evaluated prediagnosis AD use, one study assessed prediagnosis and postdiagnosis AD use (27), and three studies (28, 32, 33) examined postdiagnosis AD use; however, none of the studies indicated the purpose of AD use. The definition of AD use and the reference group varied by studies. Seven studies (24–26, 28–30, 32) compared AD use versus no use, and two studies (31, 34) compared high dose versus no use. Two studies evaluated AD use only among those who purchased ADs at least once: Boursi and colleagues (27) compared continuous use versus past use, and Shoval and colleagues (33) compared good adherence versus nonadherence.

#### Main Outcomes

Six studies (24–26, 30, 31, 34) evaluated the incidence of lung cancer as the primary outcome of their study. Four studies (27, 28, 32, 33) examined overall survival, and one study (29) examined cancer-specific survival as the primary outcome. The incidence of lung cancer was ascertained from the population-based cancer registry (*n* = 2; refs. 30, 31), medical records (*n* = 4; refs. 24–26, 34), or catastrophic illness certificate (*n* = 1; refs. 34). The deaths were ascertained from medical records (*n* = 3; refs. 27, 28, 32) or government vital statistics, including the U.S. National Death Index (*n* = 2; refs. 29, 33). For a lung cancer risk outcome, 1,801 lung cancer cases were observed over 4–14 years of follow-up periods from 840,421 participants in two retrospective cohort studies, and 69,017 lung cancer cases and 286,893 controls were matched and compared in four case–control studies. For a survival outcome, a total of 3,788 deaths, including 647 lung cancer-specific deaths, were observed from 4,554 lung cancer survivors in five retrospective cohort studies.

### Quality of Included Studies

The result of the quality assessment of selected studies was reported in Table 2, showing good (*n* = 5; refs. 27, 29, 31, 33, 34) to fair (*n* = 6; refs. 24–26, 28, 30, 32) quality.

**TABLE 2:** Methodologic quality assessment using the Newcastle-Ottawa Scale for observational studies

	Assessment domain									Total score*
	Selection				Comparability		Outcomes/Exposure			
Author, year	A	B	C	D	E	F	G	H	I	
***Cohort study***										
Boursi 2018	*	*	–	–	*	*	*	*	*	7
Haukka 2010	*	*	*	*	–	*	*	–	*	7
Karim 2019	–	*	–	–	–	*	*	*	*	5
Knekt 1996	*	*	*	–	–	–	*	*	*	6
Lohinai 2016	–	*	–	–	–	–	*	*	*	4
Shoval 2019	*	*	*	–	*	*	*	*	*	8
Zingone 2017	*	*	–	–	*	*	*	*	*	7
***Case--control study***										
Boursi 2015	–	*	*	*	*	*	–	*	–	6
Tsai 2019	*	*	*	*	*	*	–	*	–	7
Toh 2007	–	*	*	*	*	*	–	*	–	6
Walker 2011	–	*	*	*	*	*	–	*	–	6

NOTE: “-“ = criteria not met; “*” = one point for meeting criteria; Overall score: 0–3 points = poor quality; 4–6 points = fair quality; 7–9 points = good quality. ***Criteria for cohort studies*.** Selection (maximum 4 stars): A = Representativeness of the exposed cohort. B = Selection of the nonexposed cohort. C = Ascertainment of exposure. D = Demonstration that outcome of interest was not present at start of study. Comparability (maximum 2 stars): Comparability of cohorts based on the design or analysis. E = The study controls for age, gender, and smoking. F = The study controls for other factors using multivariable analysis. Outcomes (maximum 3 stars): G = Ascertainment of outcome. H = Was follow-up long enough for outcomes to occur. I = Adequacy of follow-up of cohorts. ***Criteria for case--control studies*.** Selection (maximum 4 stars): A = Is the case definition adequate? B = Representativeness of the cases. C = Selection of representative controls. D = Definition of controls. Comparability (maximum 2 stars): Comparability of cases and controls based on the design or analysis. E = The study controls for age, sex, and smoking. F = The study controls for other factors using matching or multivariable analysis. Outcomes (maximum 3 stars): G = Ascertainment of exposure. H = Same method of ascertainment for cases and controls. I = Same nonresponse rate for both groups.

### AD Use and Lung Cancer Risk

**FIGURE 2:**
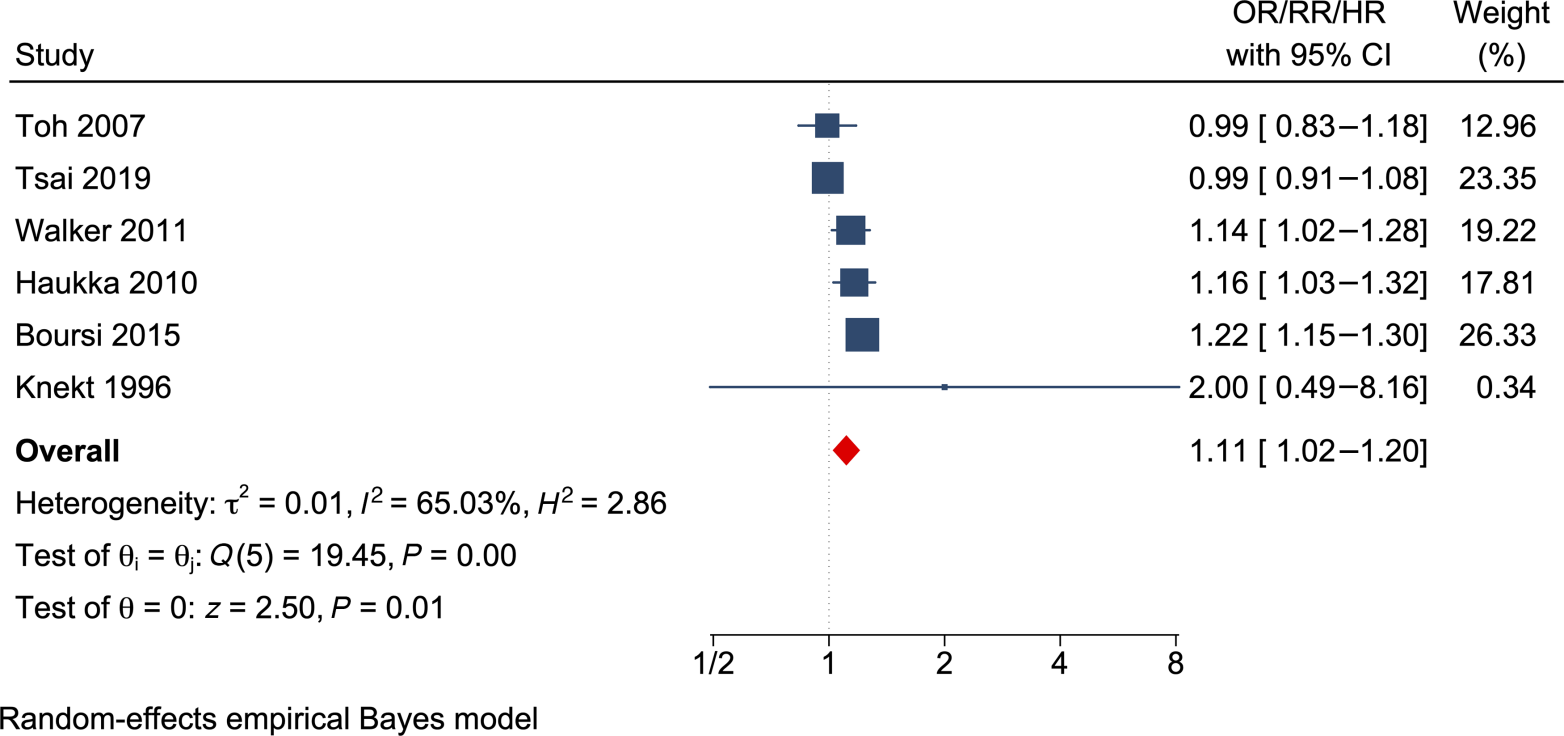
Forest plot showing the pooled effects of AD use on lung cancer. Blue square and horizontal lines represent relative risks (RR) and 95% CI for each study. The blue boxes reflect the statistical weight of the study. The dotted vertical line indicates the line of no effect. The red diamond represents the pooled RR with its 95% CI.

As shown in the forest plot (Fig. 2), the meta-analysis for lung cancer incidence included six publications: two retrospective cohort studies (30, 31), two matched case–control studies (24, 34), and two nested case–control studies (25, 26). The result showed that AD use increased lung cancer risk by 11% (RR = 1.11; 95% CI = 1.02–1.20; *I*^2^ = 65.03%; *n* = 6). Further subgroup analysis by the type of ADs displayed in Table 3 showed that SNRIs were associated with an increased risk of lung cancer risk (RR = 1.38; 95% CI = 1.07–1.78; *n* = 2); however, SSRIs (RR = 1.05; 95% CI = 0.91–1.21; *n* = 4) and TCAs (RR = 1.09; 95% CI = 0.95–1.26; *n* = 4) were not associated with an increased lung cancer risk.

**TABLE 3:** Subgroup meta-analysis evaluating AD use and lung cancer risk and survival

	Lung cancer risk			Overall survival		
	No. of studies	RR/OR/HR (95% CI)	*I* ^2^ (%)	No. of studies	RR/OR/HR (95% CI)	*I* ^2^ (%)
**Types of drugs**						
SSRI	4	1.05 (0.91–1.21)	73.3	3	1.09 (0.66–1.80)	80.5
SNRI	2	**1.38 (1.07–1.78)**	0	NA		
TCA	4	1.09 (0.95–1.26)	75.4	2	2.32 (0.35–15.52)	80.5
**Study location**						
Hungary	NA			1	0.81 (0.56–1.16)	0
Israel	NA			1	**0.79 (0.64–0.98)**	0
Finland	2	**1.17 (1.03–1.32)**	0	NA		
United Kingdom	3	**1.14 (1.02–1.27)**	64.9	1	**1.51 (1.32–1.73)**	0
United States	NA			1	1.21 (0.67–2.19)	0
Taiwan	1	0.99 (0.92–1.08)	0	NA		
**Study design**						
Case–control	2	1.06 (0.92–1.21)	73.2	NA		
Retrospective cohort	2	**1.17 (1.03–1.32)**	0	4	1.04 (0.68–1.58)	90.1
Nested case–control	2	1.12 (0.91–1.37)	80.6	NA		
**Study quality**						
Score ≥7	2	1.07 (0.91–1.25)	76.9	2	1.10 (0.58–2.07)	96.0
Score < 7	4	**1.15 (1.01–1.22)**	51.6	2	0.92 (0.64–1.33)	22.1
**Timing of antidepressant use**						
Before diagnosis	6	**1.11 (1.01–1.22)**	74.3	NA		
Before and after diagnosis	NA			1	**1.51 (1.32–1.72)**	0
After diagnosis	NA			3	**0.82 (0.69–0.98)**	0
**Comparison group**						
High dose vs. no use	2	1.07 (0.91–1.25)	76.9	NA		
Use vs. no use	4	**1.14 (1.03–1.27)**	51.6	2	0.92 (0.64–1.33)	22.1
Current use vs. past use	NA			1	**1.51 (1.32–1.73)**	0
Good adherence vs. No adherence	NA			1	**0.79 (0.64–0.98)**	0

NOTE: Significant findings are bold. Abbreviations: NA, not applicable; SNRI, serotonin and norepinephrine reuptake inhibitor; SSRI, selective serotonin reuptake inhibitor; TCA, tricyclic antidepressants.

### AD Use and Survival

**FIGURE 3:**
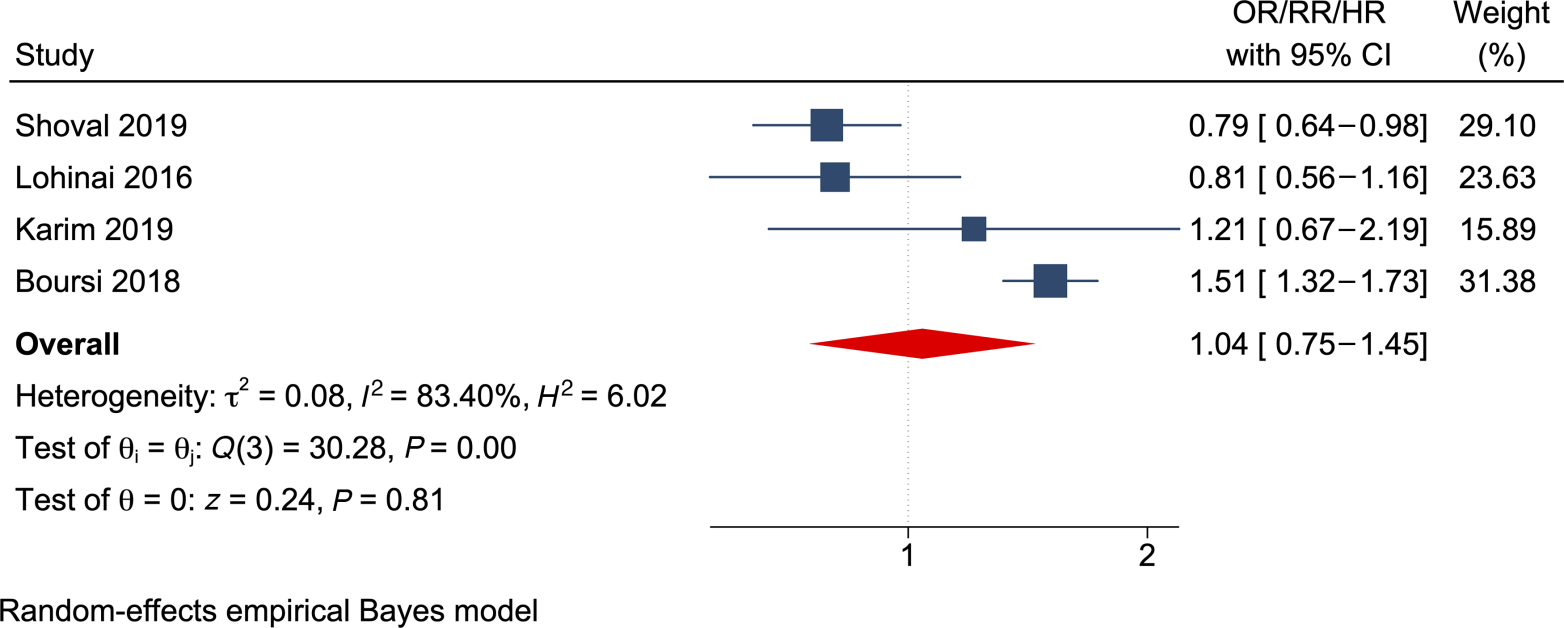
Forest plot showing the pooled effects of AD use on survival among lung cancer survivors. Blue square and horizontal lines represent relative risks (RR) and 95% CI for each study. The blue boxes reflect the statistical weight of the study. The dotted vertical line indicates the line of no effect. The red diamond represents the pooled RR with its 95% CI.

Four studies (27, 28, 32, 33) reported overall survival as the primary outcome of their research, while one study (29) reported the result of lung cancer–specific survival. Overall survival was not associated with AD use (RR = 1.04; 95% CI = 0.75–1.45; *I*^2^ = 83.40%; *n* = 4), as shown in Fig. 3. The results from subgroup analysis for overall survival in Table 3 showed that neither SSRIs (RR = 1.09; 95% CI = 0.66–1.80; *n* = 2) nor TCAs (RR = 2.32; 95% CI = 0.35–15.52; *n* = 2) were associated with survival. None of the studies examined SNRIs. Furthermore, the results varied by the definition of exposure and the reference group. For example, Shoval and colleagues (33) compared AD users who were good adherents to those who were nonadherents, while Boursi and colleagues (27) compared continuous AD users with past AD users. Continuous AD use from prediagnosis to postdiagnosis was associated with lower survival (27), while postdiagnosis AD use was associated with increased survival (RR = 0.82; 95% CI = 0.69–0.98; *n* = 3). Studies conducted in Hungary (32) and Israel (33) showed positive associations between survival and AD use, while studies from the United Kingdom (27) and the United States (28) implied inverse associations. One study (29) reported increased lung cancer–specific survival with AD use (RR = 0.68; 95% CI = 0.49–0.95).

### Publication Bias

Funnel plots (Supplementary Fig. S1) do not indicate noteworthy evidence of publication bias. The *P* values from Egger tests were 0.676 for studies included in the analysis of lung cancer risk and 0.827 for the analysis of overall survival.

## Discussion

To our knowledge, the current study is the first meta-analysis of observational studies examining the associations of AD use with lung cancer risk and survival. Our study demonstrates that AD use could increase the risk of lung cancer by 11%, with the most increased risk with SNRI use. Among patients with lung cancer, AD use was not associated with survival.

First, AD use increased the risk of lung cancer, which is somewhat contrasting with the results from previous meta-analyses in other cancers. Meta-analyses for breast cancer (35), colon cancer (36), and epithelial ovarian cancer (15) showed that AD use was not associated with an increased cancer risk. In contrast, another meta-analysis showed that AD use was associated with a reduced risk of colon cancer (14). The discrepancy in the results may be due to the differences in the types of ADs, cancer type, study design, and the number of studies included in the meta-analysis. For example, the subgroup analysis of Li and colleagues (35) including only the results from cohort studies showed an increased breast cancer risk with AD use, which is consistent with our results. The results varied by type of ADs. SSRI use was not associated with colon cancer risk (36), but TCA use was associated with a lower risk (14). In addition, as shown in our subgroup analysis, the definition and timing of AD use and the study location could be the source of heterogeneity in observed results. Considering that AD use was linked to an increased risk for stroke (RR = 1.41; 95% CI = 1.13–1.69; *n* = 31; ref. 37) and dementia (38) with both SSRIs (RR = 1.75; 95% CI = 1.03–2.96, *n* = 5) and TCAs (RR = 2.13; 95% CI = 1.43–3.18; *n* = 4) from the meta-analysis, health professionals need to be aware of the potential toxicity with long-term use of ADs.

In our analysis, only SNRIs showed an increased risk for lung cancer. SNRIs are commonly prescribed for depressive disorders, anxiety, and chronic pain for their ability to inhibit the reuptake of serotonin and norepinephrine, increasing the concentration of these neurotransmitters in the brain (16, 39). Because the management of depressive disorders, anxiety, and chronic pain may require long-term treatments and high cumulative exposure to SNRI use, the genotoxic effect of these drugs has been evaluated in several preclinical experiments. Treatment in mice with duloxetine, a commonly prescribed SNRI, led to increased oxidative DNA damage (40) and production of micronucleated erythrocytes (41), a key indicator of genetic damage. In addition, long-term administration of duloxetine to female mice resulted in the formation of liver tumors (42). Exposure of human peripheral blood lymphocytes to venlafaxine, another SNRI, generated chromosomal abnormalities such as chromatid breaks and sister chromatid unions producing an elevated concentration of micronucleated cells (43). Collectively, these studies suggest a potential role of SNRIs in increasing cancer risk by inducing DNA damage; however, considering that our results were based on a small number of studies, more studies with SNRIs and lung cancer are needed to confirm our findings.

Although many preclinical studies have reported tumor suppressive effects of AD use (8–12), our meta-analysis for overall survival from four observational epidemiologic studies did not show that AD use with either SSRIs or TCAs increased survival among lung cancer survivors, which is consistent with Chen and colleagues meta-analysis (14) for colorectal cancer reporting no associations between AD use and overall survival. Among the aforementioned four studies included in the analysis of survival outcome, one study (33) suggested that mortality could be reduced by 21% in patients with lung cancer with high adherence to AD prescription compared with the least adherent group, which may be due to increased compliance to cancer treatments with the management of depression with AD use in patients with cancer (44). When the authors restricted the analysis to those with the diagnosis of depression, the effect size was greater than the analysis with a whole sample (HR = 0.52 vs. HR = 0.79). For lung cancer–specific survival, meta-analysis was not possible because only one study reported this outcome. Zingone and colleagues study (29) reported the result with three different ADs (SSRIs, SNRIs, and TCAs) among 1,097 patients with lung cancer with 5-year follow-up, among which only TCA use before cancer diagnosis was associated with improved survival, which is supported by Chen and colleagues study, showing only TCA use being associated with a lower colon cancer risk (14), and many aforementioned preclinical studies. More epidemiologic research is needed to precisely estimate the association of AD use with lung cancer–specific survival.

There are several limitations of the current analysis that should be considered. First, there was substantial heterogeneity in key study features among the published reports, including study setting, study populations, the definition of exposure, and study designs, which probably contributed to the summary measures of the meta-analysis. Second, the number of studies included in the current meta-analysis is relatively small, so the meta-analysis effect estimates are estimated with low precision. Therefore, the results of our study should be interpreted cautiously because the estimation of heterogeneity can be challenging in meta-analyses where the number of studies included in the analysis is small (*n* ≤ 5; ref. 45). As more studies are published in the future, an updated meta-analysis is warranted. Third, although we extracted the results from the multivariable-adjusted analysis, there may be residual confounding because many studies did not include important confounders such as smoking, depression, and cancer treatment (46). Smoking history/status, the most significant risk factor for lung cancer, was adjusted in seven studies (24, 26, 27, 29, 30, 33, 34); however, the number of pack-years was adjusted in only one study (29). As the dose–response relationship between pack-years of smoking and lung cancer outcomes is well established (47), any residual confounding by smoking could influence the outcome of the current study. In addition, the conditions in which AD use was indicated were not specified. Although ADs are primarily prescribed to treat major depressive disorders, they are also used to manage anxiety disorders, chronic pain, and smoking cessation. Because the presence of depression directly affects the incidence of cancer and negatively impacts cancer prognosis, any residual confounding by coexisting depression could alter the result of the current analysis (44). In cases where ADs, such as bupropion or nortriptyline, are prescribed for smoking cessation, changes in cancer-related behaviors need to be monitored to assess lung cancer risk more accurately (17). Furthermore, the prognosis of lung cancer varies widely among tumor subtypes, disease stages, and treatment modalities. For example, a significant difference exists between the 5-year survival rate for NSCLC (26%) and SCLC (7%), which is more strongly associated with smoking (3). Only three studies either restricted analysis to patients with histologically confirmed cases or adjusted for tumor subtypes (28, 29, 32). Therefore, survival data also need to be interpreted with caution, because they were not adjusted for variables such as duration of cancer treatment or availability of targeted therapy. Future studies to evaluate the effect of ADs on lung cancer survival would benefit from including the number of pack-years, an indication for AD prescription, tumor subtypes, and cancer treatment.

There are several strengths of the current analysis to be noted. First, this study represents the first meta-analysis of lung cancer risk and survival with AD use. Next, in nine (24–28, 31–34) out of 11 studies, AD use was obtained from medical/prescription records. Cancer diagnosis and death were also accessed from medical records or national/governmental vital statistics, which are less likely to be influenced by recall bias. However, exposure misclassification is still possible because adherence was only assessed in one study. Nonetheless, misclassification was more likely to be nondifferential, biasing the results toward the null. Finally, most studies were based on a large population-based sample with a sufficient follow-up period, which increases the generalizability of study findings.

## Conclusions

In summary, the number of studies assessing the associations between AD use and lung cancer risk and survival is deficient despite the high prevalence of depression and high usage of ADs in people with lung cancer. This first meta-analysis for lung cancer suggested that AD use could increase the risk of lung cancer. To increase the evidence, well-designed prospective cohort studies are needed, where commonly used ADs (SSRIs, SNRIs, and TCAs) are evaluated on cancer outcomes in individuals with lung cancer and with or without a diagnosis of depression.

## Supplementary Material

Supplementary Table S1 Full Search Strategies

Supplementary Table S2 Criteria for inclusion and exclusion of studies

Supplementary Figure S1 Supplementary Figure 1: A funnel plot for the meta-analysis between antidepressant use and lung cancer risk and mortality.
