# Supplementary material for: Antidepressant Use and Lung Cancer Risk and Survival: A Meta-analysis of Observational Studies
Source: Cancer Res Commun. 2023 Jun 12;3(6):1013–25. doi: 10.1158/2767-9764.CRC-23-0003 (PMC10259481; doi:10.1158/2767-9764.CRC-23-0003)
Supplement: Supplementary Table S1 — Full Search Strategies [file crc-23-0003-s01.docx]

**Supplementary Table S1. Full Search Strategies** (Interface – EBSCOhost, Database -MEDLINE)

| **#** | **Query** | **Limiters/Expanders** | **Results** |
| --- | --- | --- | --- |
| S1 | lung cancer or lung neoplasms or lung tumo* or lung adenocarcinoma or NSCLC or SCLC | Expanders  - Apply equivalent subjects  Search modes  - Find all my search terms | 430,156 |
| S2 | (Antidepressants or anti-depressants or antidepressant medication or SSRI or selective serotonin reuptake inhibitors or tricyclic antidepressant* or TCA or serotonin-norepinephrine reuptake inhibitor* or SNRI) OR (Amitriptyline or Amoxapine or Benactyzine or Bupropion or Citalopram or Clorgyline or Clovoxamine or Deanol or Desipramine or Dothiepin or Doxepin or Fluvoxamine ) OR (imipramine or Tofranil or Iprindole or Lofepramine or Mianserin or Nialamide or Nortriptyline or Opipramol or Paroxetine or Phenelzine or Pizotypline or (Vilazodone Hydrochloride) or Viloxazine or Sulpiride or Sertraline or Rolipram ) | Expanders  - Apply equivalent subjects  Search modes  - Find all my search terms | 129,996 |
| S3 | S1 & S2 | Expanders  - Apply equivalent subjects  Search modes  - Find all my search terms | 415 |
| S4 | (Mortality or mortality rate or death or death rate) OR (survival rate or survival outcomes or clinical outcomes) OR (incidence or risk) | Expanders  - Apply equivalent subjects  Search modes  - Find all my search terms | 5,935,304 |
| S5 | S3 AND S4 | Expanders  - Apply equivalent subjects  Search modes  - Find all my search terms | 143 |
| S6 | S3 AND S4 | Limiters  - Scholarly (Peer Reviewed) Journals  Expanders  - Apply equivalent subjects  Search modes  - Find all my search terms | 141 |
