# Supplementary material for: Antidepressant Use and Lung Cancer Risk and Survival: A Meta-analysis of Observational Studies
Source: Cancer Res Commun. 2023 Jun 12;3(6):1013–25. doi: 10.1158/2767-9764.CRC-23-0003 (PMC10259481; doi:10.1158/2767-9764.CRC-23-0003)
Supplement: Supplementary Table S2 — Criteria for inclusion and exclusion of studies [file crc-23-0003-s02.docx]

**Supplementary Table S2. Criteria for inclusion and exclusion of studies**

| Criteria | Description for lung cancer risk study | Description for lung cancer mortality study |
| --- | --- | --- |
| Patients | Adults, age ≥ 18 years, men and women | Adult lung cancer survivors, age ≥ 18 years, men and women |
| Exposure | Antidepressant use before cancer diagnosis | Antidepressant use before cancer diagnosis or after cancer diagnosis |
| Comparison/control | No/low use | No/low use |
| Outcome | Lung cancer incidence | Mortality (all-cause and/or cancer-specific) |
| Study Design | Cohort or case-control studies | Cohort studies |
