# Supplementary figures and images for: Antidepressant Use and Lung Cancer Risk and Survival: A Meta-analysis of Observational Studies
Source: Cancer Res Commun. 2023 Jun 12;3(6):1013–25. doi: 10.1158/2767-9764.CRC-23-0003 (PMC10259481; doi:10.1158/2767-9764.CRC-23-0003)

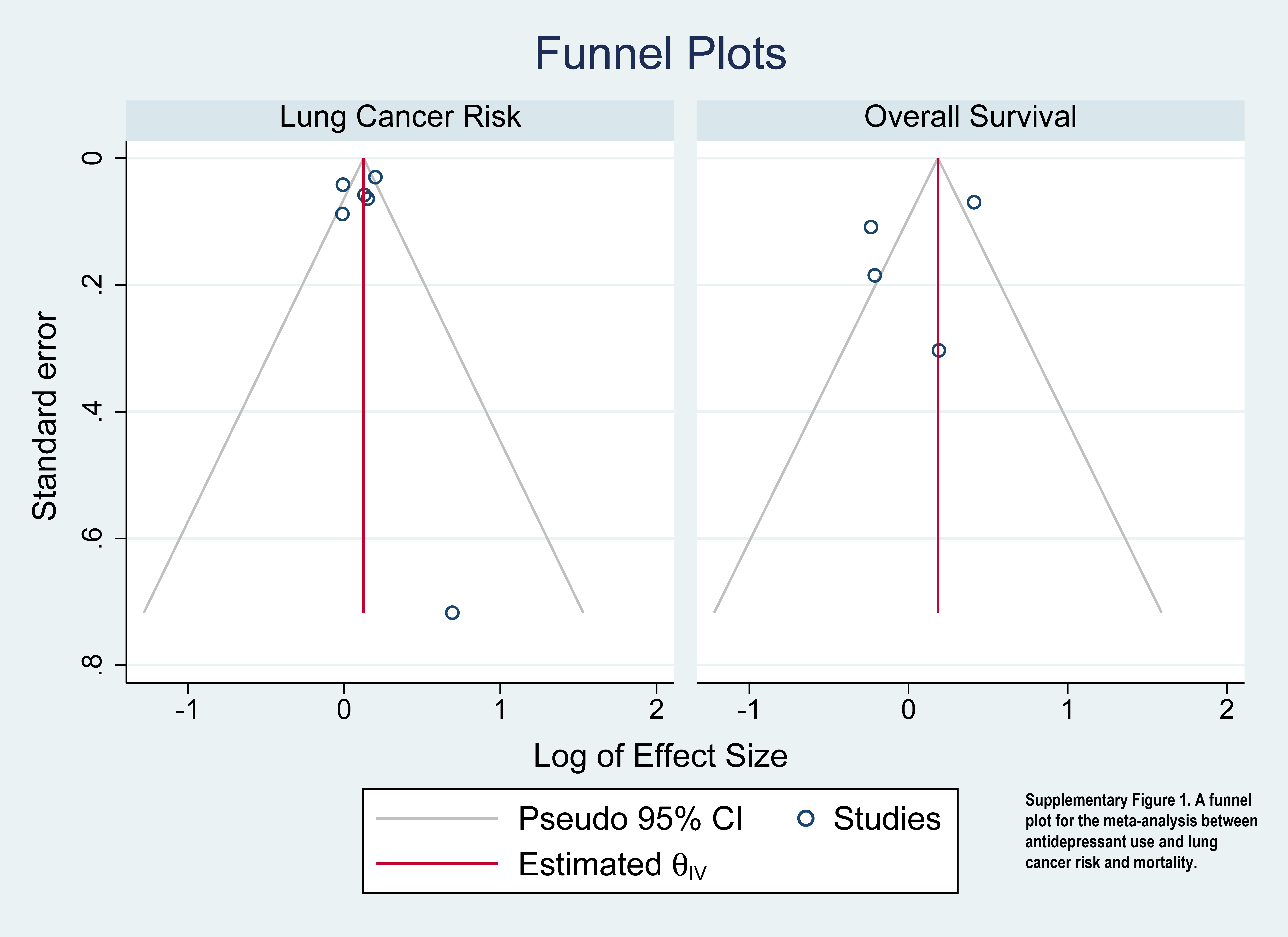

Supplement: Supplementary Figure S1 — Supplementary Figure 1: A funnel plot for the meta-analysis between antidepressant use and lung cancer risk and mortality. [file crc-23-0003-s03.png]
